# Clinical audit for occupational therapy intervention for children with autism spectrum disorder: sampling steps and sample size calculation

**DOI:** 10.1186/s13104-015-1247-0

**Published:** 2015-06-30

**Authors:** Scott Weeks, Alvin Atlas

**Affiliations:** International Centre for Allied Health Evidence (iCAHE), Sansom Institute, University of South Australia, Adelaide, Australia

**Keywords:** Sampling frame, A priori, Sample size calculation, Clinical audit, Autism spectrum disorder

## Abstract

A priori sample size calculations are used to determine the adequate sample size to estimate the prevalence of the target population with good precision. However, published audits rarely report a priori calculations for their sample size. This article discusses a process in health services delivery mapping to generate a comprehensive sampling frame, which was used to calculate an a priori sample size for a targeted clinical record audit. We describe how we approached methodological and definitional issues in the following steps: (1) target population definition, (2) sampling frame construction, and (3) a priori sample size calculation. We recommend this process for clinicians, researchers, or policy makers when detailed information on a reference population is unavailable.

**Electronic supplementary material:**

The online version of this article (doi:10.1186/s13104-015-1247-0) contains supplementary material, which is available to authorized users.

## Background

Sampling is the process of selecting a sub-group of individuals, or observations that can accurately represent the characteristics of the entire target population [1]. Irrespective of the type of study design being applied, probability sampling, which employs random selection of participants, is the preeminent method for obtaining a representative sample and minimising sampling bias. However, to apply probability sampling, it is essential that each unit of the target population is known and has an equal chance of being selected. Therefore, before any sample can be determined, it is essential to have a clear definition of population parameters, and the purpose and characteristics for evaluation [1, 2].

A priori sample size calculation is used to determine the adequate sample size to estimate the prevalence of the target population with good precision [3]. Concerning audits, Fournel et al. [4] indicates that published audits rarely report a priori calculations for their sample size. This paper (1) describes our experiences in health services delivery mapping to generate an up-to-date and comprehensive sampling frame, and (2) outlines the process of calculating an a priori sample size for a targeted clinical record audit.

**Figure 1:**
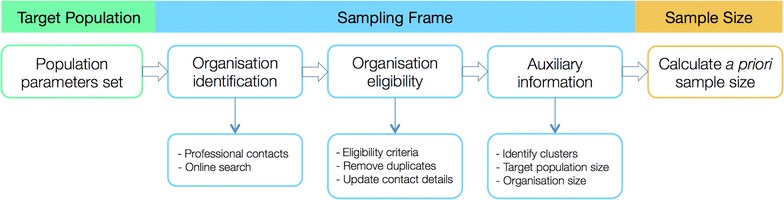
Process flow diagram to calculate a priori sample size.

The larger purpose for our research is to describe the range of classifications, assessments, interventions and outcomes measures, being used and recorded by South Australian paediatric occupational therapists, when treating sensory difficulties in children with autism spectrum disorder (ASD). We believe that this is best collected using an audit of current practice. From this information, we intend to understand the various ways that sensory assessments and interventions are being administered in community-clinic-based settings, how treatment decisions are being made, the relationship between treatment decisions and key demographic variables, the frequency of intervention and compliance with prescribed guidelines of various types of interventions, and what outcome measures are being used to determine the effectiveness of these interventions. This paper reports on how we tackled the methodological and definitional issues in the following three steps (Figure 1).

## Methods

### Step 1: Defining the target population

The first stage of the sampling process was to clearly define our target population [1, 5]. We identified our population as the clinical (patient) records of children with ASD who have received assessment and intervention for sensory difficulties by South Australian paediatric occupational therapists. Moreover, we are specifically interested in children who have received an ASD diagnosis based on criteria outlined in the *Diagnostic and Statistical Manual of Mental Disorders, 5th edition* (DSM-5) [6] and who were aged 12 years or less when commencing therapy. Detailed clinical records must have been maintained by occupational therapists that documented the course of assessment and intervention over multiple occasions of service, within an extended episode of care. More detailed information on the population parameters can be obtained from the authors.

### Step 2: Constructing the sampling frame

A comprehensive sampling frame is necessary for probability sampling. A sampling frame is a comprehensive list of the target population [5] from which the investigator can select individuals or units into the study [1]. Service user and service provider lists can be untrustworthy and problematic for audits, due to incomplete registers, inconsistency in the uptake to these registers, and variation in the documentation of the services provided [2]. We have previously highlighted the difficulties associated with obtaining a non-biased sample of children with ASD because of the lack of a national register [7]. It is not the intention of this paper to advocate for the development of a register, but to consider the challenges we faced in accessing a reliable sampling frame for an esoteric area of research. Our initial barrier was the inability to locate a comprehensive service provider list that identified organisations providing sensory-based occupational therapy intervention to children with ASD.

#### Identification of potential organisations

It is advised to use several sources to identify the target population when developing a sampling frame [2]. In the first instance, a primary list of potential organisations was generated by the authors on the basis of their collective clinical, academic and research experience. This list was expanded through online searches of publically listed information until saturation of organisation names had been reached. The research team determined that organisations could be assigned to clusters based on (1) the health sector they were situated and (2) geographical location (metropolitan and country regions), as it was anticipated that differences may exist in service delivery across these clusters. The initial clustering by sector that the research team anticipated to be important for stratification included (1) public services, (2) non-government organisations (NGO), (3) private organisations and (4) special schools. Understanding differences in service delivery was an important consideration in our sampling frame.

#### Eligibility of organisations

Identifying organisations that meet the eligibility criteria, removing duplicate listings and ensuring the currency of contact information are vital components to constructing a robust sampling frame [5]. A telephone survey was conducted to the primary contact list to identify service eligibility for the study. This process concurrently confirmed duplicate listings and current contact details. Organisations that met the eligibility criteria were emailed the primary contact list. Using a snowball sampling approach, individuals representing organisations on the list were asked to recommend other organisations not identified through the primary search. This continued until saturation was reached.

#### Auxiliary information

DiGaetano [5] recommends obtaining additional information beyond the identification of names and contact details, as it can be useful for the purposes of sampling and weighting. To this end, eligible organisations were emailed an online questionnaire to elucidate auxiliary information. The combined results from the online questionnaire and telephone survey were used to identify congruencies and divergence in service delivery between organisations, health sectors and geographical locations, to further inform the aforementioned clustering. Moreover, information was gathered to estimate how many children diagnosed with ASD based on DSM-5 criteria (ASD/DSM-5) had received occupational therapy intervention per organisation, and what proportion of these children subsequently received intervention for sensory difficulties. Organisations that could not provide this information were asked to estimate how many children with ASD (based on any criteria) they treated per month and confirm the number of full-time equivalent occupational therapists employed at their organisation. This information was used to provide an estimate of the total number of clinical records from which a sample would be drawn, as well as assign a weighting to each organisation that could not estimate specific numbers of ASD/DSM-5.

### Step 3: Sample size calculation

South Australian organisations that provide intervention for sensory difficulties to children with ASD/DSM-5 were the source of the patient records that will be used for this audit. In clinical audit, evaluating whether a particular intervention is given to the patient with reference to the standard, the required number of patients is calculated by the desired precision level of the compliance rate. In this study, the calculation of the required number of subjects depended on the expected rate of sensory-based intervention given and the desired precision of the estimate with a set confidence interval.

Sample size calculation formula: For 95% confidence level and +5% accuracy, the sample size would be calculated using the formula:

$${\text{n }} = \, \left[ {{\text{z}}^{ 2} \times {\text{ N }} \times \, \left( {{\text{p}}\left( { 1- {\text{p}}} \right)} \right)} \right] \, \Big/ \, \left[ {\left( {0.0 5^{ 2} \times {\text{ N}}} \right) \, + \, \left( {{\text{z}}^{ 2} \times \, \left( {{\text{p }}\left( { 1- {\text{p}}} \right)} \right)} \right)} \right]$$

where n is the sample size, z^2^ is the abscissa of the normal curve that cuts off an area at the tails, N is the population size, and p is the estimated proportion that the intervention is being given to children with ASD. The sample size will then be allocated to the clinics using proportional allocation [8]. Using the sample size sufficient for a 95% level of confidence and 5% accuracy, it can be stated that there is 95% certainty that the true value of the estimated proportion of the sensory-based intervention that is being given to children with ASD is between 90 and 100%.

## Results

### Constructing the sampling frame

#### Identification of potential organisations

A total of 174 potential organisations were identified from which to generate the primary contact list. This included 26 organisations through research team contacts and 148 organisations through online searching. A further three potential organisations were identified through the snowball sampling approach; however none met the inclusion criteria. The snowballing sampling approach also identified an incorrect classification, where one organisation listed as a public service on the preliminary contact list needed to be amended to NGO.

#### Eligibility of organisations

A total of 33 organisations met the eligibility criteria for the proposed clinical record audit. When clustering for sector, seven were public, 23 were private, and three were NGOs. When clustering based on geographical location, 24 were metropolitan organisations and nine were country organisations. It was reported that some organisations based in metropolitan Adelaide offer services to country regions, and conversely, some country children travel to metropolitan organisations for intervention. Consequently, the clinical records of children with ASD living in country locations may be kept at metropolitan sites. On this basis, we determined that geography was not a useful approach to determine clusters. Instead, child demographics and metropolitan and country service provision stratification would be most reliably extracted during the clinical record audit.

#### Auxiliary information

It became apparent through the development of the sampling frame that the initial clustering by sector was also invalid due to the current funding climate and when measured against the research aims. With the inception of the National Disability Insurance Scheme (NDIS) as a prominent means of funding services within our target population [9], there are organisations within public and NGO sectors that now provide assessment and intervention in the same manner as private organisations. On the other hand, some organisations that provide types of sensory assessment and/or intervention to children with ASD did not fully meet the eligibility criteria of the audit. Moreover, not all children with ASD will be receiving NDIS funding, as funding is determined by the level of severity of ASD. In these cases, children could attend private occupational therapy practices for intervention based on other types of funding arrangements (i.e., parent’s out-of pocket expense, Medicare, private health insurance). Likewise, children could attend public and NGO practices based on parent out-of pocket expenses. Nonetheless, methods of funding did not alter the reported service provision between the eligible organisations that met the eligibility criteria for this study. This process highlighted a new set of clusters in the service provision for the sensory needs in children with ASD in South Australia (Additional file 1: Table S1). This organic development resulted in only one cluster being eligible for inclusion into our proposed audit, which comprised of a combination of private, NGO and public sector organisations. Lastly, the consensus from organisation report was that approximately 95% of children with ASD who engage occupational therapy services subsequently receive assessment and intervention for sensory difficulties. This proportion is consistent with the literature, which shows that up to 95% of children with ASD experience at least one sensory processing difficulty [10].

### Sample size calculation

We estimated conservatively, that there were 421 children in total, with a diagnosis of ASD/DSM-5 who have received occupational therapy intervention through South Australian organisations situated in cluster 3. Moreover, we estimated that 95% of these patients would be given intervention in some form to address their sensory difficulties. We consequently estimated that our required baseline sample size would be 63 clinical records.

Sample size calculation

$$\begin{aligned} {\text{n }} &= {\text{ [z}}^{2} \times {\text{N}} \times ({\rm p(1 - p)}) ] / [ ( 0. 0 5^{2} \times {\text{N)}}{ + } ( {\text{z}}^{2} \times ({\rm p(1 - p)}) ) ]\hfill \\ &= [1.96^{2} \times 421 \times (0.95(1 - 0.95))]/[(0.05^{2} \times 421) + (1.96^{2} \times (0.95(1 - 0.95)))] \hfill \\ &= { 62}. 1 9\sim 6 3 {\text{ subjects}} \hfill \\ \end{aligned}$$

However, we insured the robustness of our sample, by inflating the baseline sample size by 10%, to account for incomplete or missing data. Thus, the final audit sample would be at least 70 clinical records. Since the data collection points identified in our second step (places where children with ASD receive occupational therapy intervention) have different numbers of patients attending, proportional allocation based on the number of patients per clinic would need to be used to determine the number of sample to be taken per clinic (Additional file 2: Table S2).

## Conclusion

This approach is the first that we know of, in the world of ASD research that has sought to produce a comprehensive audit sampling frame based on clinic unit and inclusion criteria for children with ASD, who receive assessment and intervention for sensory difficulties, by occupational therapists, in a defined geographic area. The intention of this paper was to report on the process that clinicians, researchers, or policy makers should take when calculating an a priori sample size, with robust methodology, in an area where there are no reliable reference population estimates. This could pertain to audits, but also to observational studies or experimental studies related to effectiveness of practice.

## Additional files

Additional file 1:
**Table S1.** Identified service provision clusters. Additional file 2:
**Table S2.** Proportional allocation of sample per organization size.

## Abbreviations

ASD: autism spectrum disorder
DSM-5: diagnostic and statistical manual of mental disorders
NGO: non-government organisations
NDIS: National Disability Insurance Scheme
