# Supplementary figures and images for: Clinical audit for occupational therapy intervention for children with autism spectrum disorder: sampling steps and sample size calculation
Source: BMC Res Notes. 2015 Jun 30;8:282. doi: 10.1186/s13104-015-1247-0 (PMC4644071; doi:10.1186/s13104-015-1247-0)

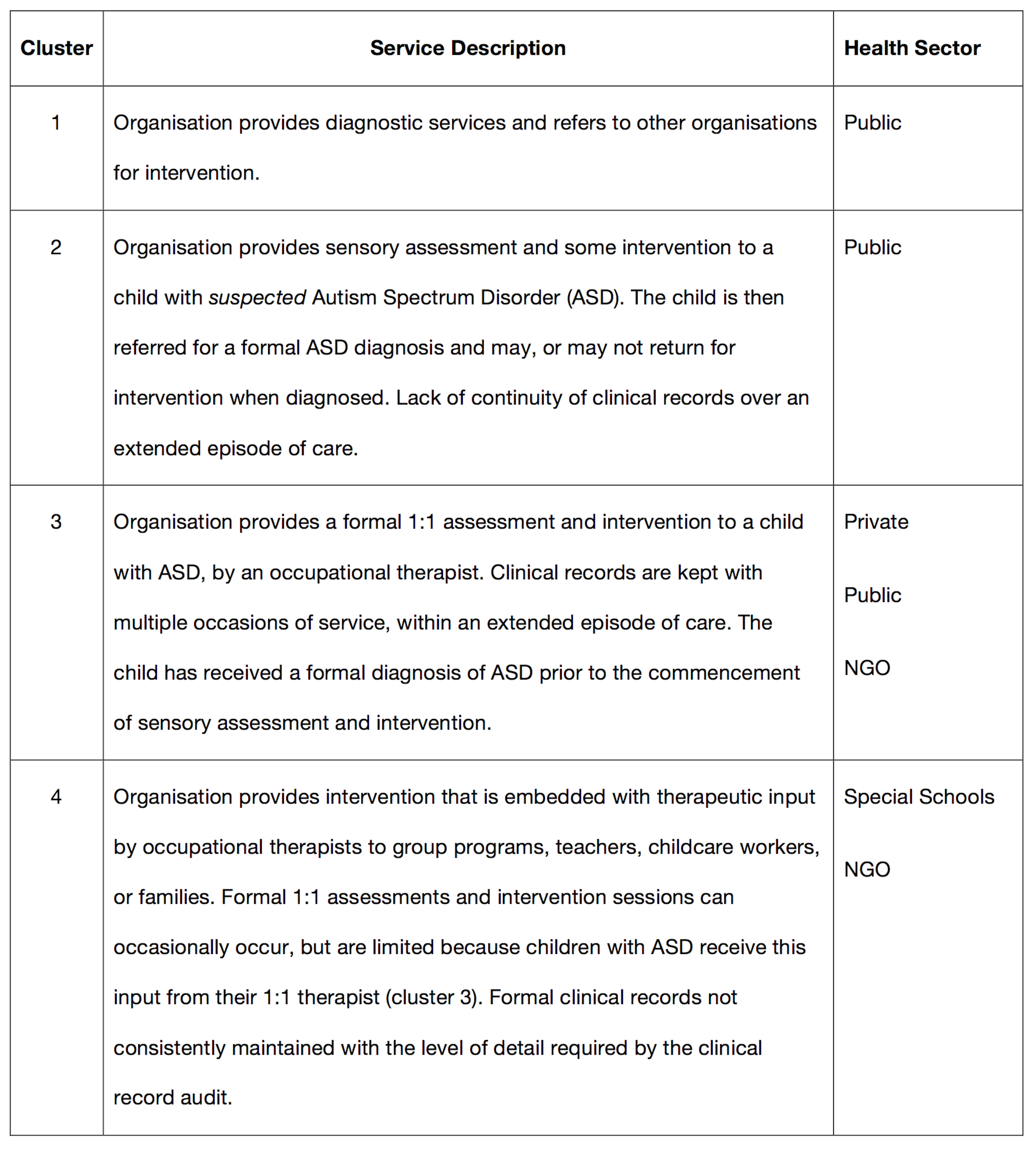

Supplement: Additional file 1: — Table S1. Identified service provision clusters. [file 13104_2015_1247_MOESM1_ESM.png]

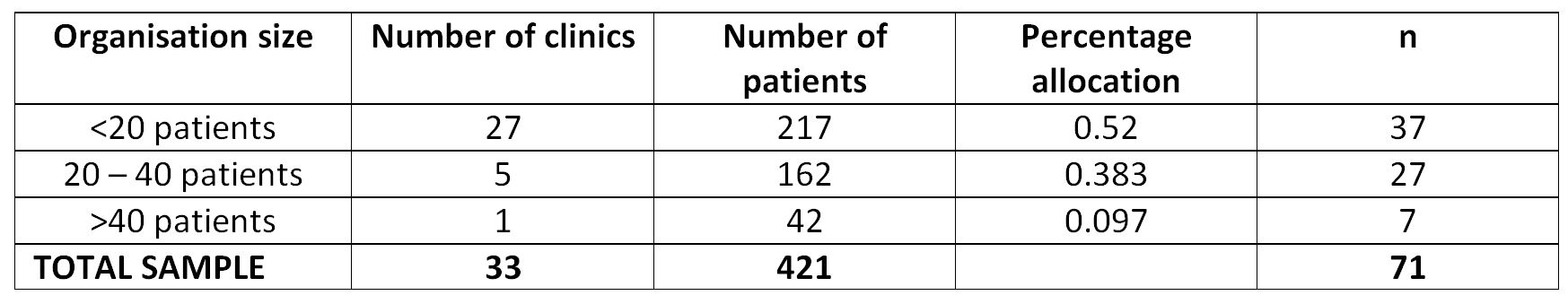

Supplement: Additional file 2: — Table S2. Proportional allocation of sample per organization size. [file 13104_2015_1247_MOESM2_ESM.jpg]
